# Association Between Glucocerebrosidase Mutations and Parkinson's Disease in Ireland

**DOI:** 10.3389/fneur.2020.00527

**Published:** 2020-06-30

**Authors:** Diana A. Olszewska, Allan McCarthy, Alexandra I. Soto-Beasley, Ronald L. Walton, Brian Magennis, Russell L. McLaughlin, Orla Hardiman, Owen A. Ross, Tim Lynch

**Affiliations:** ^1^The Dublin Neurological Institute at the Mater Misericordiae University Hospital, Dublin, Ireland; ^2^Department of Neuroscience, Mayo Clinic Jacksonville, Jacksonville, FL, United States; ^3^School of Medicine and Medical Science, University College Dublin, Dublin, Ireland; ^4^Department of Neurology, The Adelaide and Meath Hospital, Dublin, Ireland; ^5^Trinity College Dublin, Smurfit Institute of Genetics, Dublin, Ireland; ^6^Department of Neurology, Beaumont Hospital, Dublin, Ireland; ^7^Academic Unit of Neurology, Trinity College Dublin, Trinity Biomedical Sciences Institute, Dublin, Ireland; ^8^Department of Clinical Genomics, Mayo Clinic, Jacksonville, FL, United States

**Keywords:** *GBA*, glucocerebrosidase, Ireland, sequencing, Parkinson's disease

## Abstract

Multiple studies implicate heterozygous *GBA* mutations as a major genetic risk factor for Parkinson's disease (PD); however, the frequency of mutations has never been examined in PD patients from the Irish population. We prospectively recruited 314 unrelated Irish PD patients (UK Brain Bank Criteria) and 96 Irish healthy controls (without any signs or family history of parkinsonism) attending. The Dublin Neurological Institute (DNI). Complete exon *GBA* Sanger sequencing analysis with flanking intronic regions was performed. The *GBA* carrier frequency was 8.3% in PD and 3.1% in controls. We identified a number of potentially pathogenic mutations including a p.G195E substitution and a p.G377C variant, previously described in a case study of Gaucher's disease in Ireland. On genotype–phenotype assessment hallucinations, dyskinesia, and dystonia were more prevalent in *GBA*-PD. The genetic etiology of PD in Ireland differs from the continental Europe as seen with the lower *LRRK2* and higher than in most European countries *GBA* mutation frequency. Determining genetic risk factors in different ethnicities will be critical for future personalized therapeutic approach.

## Introduction

Glucocerebrosidase gene (*GBA*) encodes *B*-glucocerebrosidase enzyme hydrolyzing glucocerebroside to glucose and ceramide. While homozygous or compound heterozygous *GBA* mutations alter glucocerebrosidase activity and result in a recessive lysosomal-storage disorder, Gaucher's disease, heterozygous variants have been implicated in Parkinson's disease (PD) pathogenesis (1–3). In fact, *GBA* mutations have been identified as one of the strongest known genetic risk factors for PD (5–20% of PD patients are reported to harbor *GBA* mutations depending on ethnicity) (2). Initially, *GBA*-related PD was thought to reflect the clinical phenotype of idiopathic PD (3); however, current evidence suggests unique motor (e.g., frequent falls, freezing of gait, dyskinesia, faster progression), non-motor [autonomic symptoms, younger age at onset (2.6–0.9 year earlier age at onset)] (4), increased prevalence of rapid eye movement (REM) sleep behavior disorder (RBD) and daytime sleepiness (5) features and cognitive impairment (frequent cognitive decline and hallucinations) (5–7).

The *GBA* gene is located on chromosome 1q22 and consists of 11 coding exons. The existence of a highly homologous pseudogene (GBAP1) (sharing 96% of exonic sequence) located 16 kb downstream of the functional *GBA* gene makes *GBA* sequencing studies challenging (8). Sequencing of all exons and using long-range PCR primers specific for the functional *GBA* gene is the most reliable method for genetic screening. However, many centers continue to restrict the analysis to the most common variants or exons where most of the mutations are reported (exons 9 and 10) (9). *GBA* p.L444P and p.N370S are the two most common pathogenic substitutions among 335 reported, responsible for 50–70% of cases (1). Based on homozygous and compound heterozygous mutations resulting in specific subtypes of Gaucher's disease, *GBA* mutations can be classified as “mild” (p.N370S and p.R496H) and “severe” [p.L444P, p.D380A, p.R131C, p.D409H, p.R463C, p.R257Q, p.V394L, c.1263-1317del, and RecNciI-a recombinant allele (p.L444P-A456P-V460V)] (10). Carriers of mild mutations are reported to have 2.2-fold higher risk of PD and mean age at onset of 58.1 (±10.6), while carriers of severe mutations have 9.92- to 21.29-fold increased risk of PD and mean age at onset of 52.1 (±11.2) (10, 11). *GBA* p.E326K homozygous and compound heterozygous mutations do not cause Gaucher's disease; thus, there may be a distinct mechanism predisposing to PD in carriers of p.E326K. Until recently, believed to be a benign polymorphism, now p.E326K is an established risk factor (large meta-analyses) causing rapid motor progression of PD (β = 3.42; 95% CI, 0.66–6.17; *p* = 0.02) (12, 13), cognitive decline, and the development of RBD among those who did not have the disorder at baseline (5, 6, 11). Similarly, there is an emerging trend in the literature to classify p.T369M as a risk variant (with the reported effect size similar to that of p.E326K, baseline RBD, associated cognitive decline, and higher hazard ratio of reaching H&Y3) (4, 6, 14, 15).

The prevalence of *GBA* mutations can vary according to ethnicity (10) (e.g., mutations are common in Ashkenazi Jewish populations). Locally derived data are important for further genetic characterization, development of local guidelines, enrolment in clinical trials, and search for the disease-modifying treatments. Large Irish families, small population size of the country (Republic of Ireland population, 4.79 million in 2017, as per the most recent census) (16), and relative isolation from the continental Europe make the Irish population ideal for genetic studies. We and others reported that genetics of PD in the Irish population differs from that in the continental Europe, e.g., *LRRK2* mutations are rare (17). We have shown in a recent epidemiological study that the population structure in Ireland differs from the rest of the Europe, and this may be related to the Celtic ancestry (18). We have also demonstrated that the prevalence of another neurological condition, amyotrophic lateral sclerosis (ALS) in Ireland, differs from other European countries (19). The prevalence of *GBA* in PD and genotype-phenotype correlation has never been studied in Ireland, and we hypothesized that similarly to *LRRK2*, it may differ from that in other populations.

## Methods

### Ethical Approval

The ethical approval (1/378/1,300) was granted by the Mater Misericordiae University Hospital (MMUH), Dublin, Ireland. Informed written consent was obtained.

### Design

This is an observational cross-sectional study.

### Participants

Three hundred fourteen unrelated Irish patients over age 18 diagnosed with PD (UK Brain Bank Criteria) were recruited from a tertiary referral center, Dublin Neurological Institute (DNI), at the MMUH, Dublin, Ireland. Patient's attending the DNI reside in the Dublin city or rural areas of the country. To expand the diversity of participants, an additional notice about the study recruitment was posted on the Irish Parkinson's Association website. Secondary and atypical forms of parkinsonism were excluded. Ninety-six healthy controls (friends or spouses of patients) without any signs or family history of parkinsonism, over age 18 were recruited from the DNI.

### Sequencing and Data Analysis

Genomic DNA was isolated from peripheral blood lymphocytes using QIAmp Blood Midi Kit. Genetic analysis was performed at the Department of Neuroscience, Mayo Clinic, Jacksonville, FL, USA. All PD patients were prescreened for the most common genetic causes of autosomal dominant disease (*LRRK2, SNCA, VPS35*) and patients with young onset PD (YOPD) (age, <50) also for autosomal recessive PD (*PARKIN, PINK1, DJ1*). PD patients (*n* = 314) were tested for specific point mutations; LRRK2 p.G2019S, SNCA p. A53T, and p.A30P and VPS35 p.D620N (TaqMan Allelic Discrimination Assay, on Quant Studio System Real-Time PCR System). Multiplex ligation-dependent probe amplification (MLPA) analysis was employed to determine the dosage alterations in *SNCA*. PD patients with YOPD (*n* = 81) were investigated by Sanger sequencing of all coding exons plus 25 base pairs of exon–intron boundary of *PARKIN* (12 exons), *PINK1* (8 exons), and *DJ1* (6 exons) genes. MLPA analysis was also performed.

*GBA* sequencing of all 11 coding exons plus 25 base pairs of exon–intron boundary was performed on all patients and 96 controls and confirmed bidirectionally. PCR and primer sequences are available in Supplementary Tables 1, 2. The pathogenicity of the variants was determined based on the current literature and *in silico* tools [Polyphen v2 (20), SIFT (21), Mutation Taster (22)] (Table 1). Results are presented in Table 2. Both newer and well-established historical nomenclature (GBA protein −39 amino acids) for *GBA* variants annotation was used.

**Table 1:** Pathogenicity prediction results for *GBA* genotypes found in Irish Parkinson's disease (PD) patients and controls: pathogenic/risk variants, predicted pathogenic, and VUS genotypes.

**GBA Protein**	**GBA Protein (-39aa)**	**Genotype**	**rs #**	**Exon**	**Mutation Taster**	**Polyphen 2**	**SIFT**	**dbSNP**	**ClinVar**	**Literature**	**Irish Study**
**Pathogenic/risk GBA variants**											
E365K	E326K	G > A	rs2230288	8	Path	Benign	Tolerated	Path	Risk	Risk	Risk
T408M	T369M	C > T	rs75548401	8	Polymorphism	Benign	Tolerated	VUS	VUS	Risk	Risk
F255Y	F216Y	T > A	rs74500255	7	Path	Probably Path	Tolerated	Path	Path	Path	Path
N409S	N370S	A > G	rs76763715	9	Path	Possibly Path	Path	Path	Path	Path	Path
D448H	D409H	G > C	rs1064651	9	Path	Benign	Tolerated	Path	Path	Path	Path
L483P	RecNcil	T > C	rs421016	10	Path	Path	Path/Tolerated	Path	Path	Path	Path
A495P	Recombinant	G > C	rs368060								
V499V		G > C	rs1135675								
**Predicted pathogenic**											
G416C	G377C	G > T	No rs	9	Path	Probably Path	Path	N/A	N/A	1 patient Gaucher's	Predicted Path
**Variants of unknown significance**											
G234E	G195E	G > A	rs74462743	6	Path	Probably Path	Path	SNV	N/A	1 patient Gaucher's	VUS
R301H		G > A	rs140955685	7	Polymorphism	Benign	Tolerated	SNV	N/A		VUS
R368C		C > T	rs374306700	8	Path	Probably Path	Path	VUS	VUS		VUS

*GBA Protein (−39aa) the conventional nomenclature for GBA alleles following Human Genome Variation Society (HGVS) recommendation referring to the processed protein and excluding the 39-residue signal peptide. GBA Protein, the alternative nomenclature; Path, pathogenic; VUS, variant of unknown significance; SNV, single-nucleotide variant*.

**Table 2:** Pathogenic/risk variants and variant of unknown significance (VUS) genotypes, carrier frequency, and minor allele frequency in Irish Parkinson's disease (PD) patients and controls and comparison to ExAC and GnomAD databases.

**GBA protein**	**GBA protein (-39aa)**	**Genotype**	**rs #**	**Exon**	**PD het/homo**	**PD carrier frequency**	**PD MAF**	**Controls het/homo**	**PD carrier frequency**	**PD MAF**	**ExAC MAF**	**GnomAD MAF**
**Pathogenic/risk GBA variants**												
T408M	T369M	C > T	rs75548401	8	6/0	1.92%	0.96%	4/0	2.37%	1.18%	0.98%	0.94%
E365K	E326K	G > A	rs2230288	8	12/1	4.14%	2.23%	3/1	2.37%	1.48%	1.20%	1.23%
F255Y	F216Y	T > A	rs74500255	7	1/0	0.33%	0.16%	0/0	0%	0%	0.02%	0.00%
N409S	N370S	A > G	rs76763715	9	3/0	0.96%	0.48%	0/0	0%	0%	0.36%	0.20%
D448H	D409H	G > C	rs1064651	9	1/0	0.32%	0.16%	0/0	0%	0%	0.01%	0.02%
L483P	RecNcilrecombinant	T > C	rs421016	10	3/0	0.96%	0.48%	0/0	0%	0%	0.39%	0.14%
A495P		G > C	rs368060		3/0	0.96%	0.48%	0/0	0%	0%	0.01%	0.01%
V499V		G > C	rs1135675		3/0	0.96%	0.48%	0/0	0%	0%	0.02%	0.03%
**Predicted pathogenic**												
G416C	G377C	G > T	No rs	9	1/0	0.32%	0.16%	0/0	0%	0%	Not reported	Not reported
**Variants of unknown significance**												
G234E	G195E	G > A	rs74462743	6	1/0	0.32%	0.16%	0/0	0%	0%	–	0.00%
R301H		G > A	rs140955685	7	0/1	0.32%	0.32%	0/0	0%	0%	0.01%	0.02%
R368C		C > T	rs374306700	8	1/0	0.32%	0.16%	0/0	0%	0%	0.00%	0.00%

*GBA Protein (−39aa) the conventional nomenclature for GBA alleles following Human Genome Variation Society (HGVS) recommendation referring to the processed protein and excluding the 39-residue signal peptide. GBA Protein, the alternative nomenclature; MAF, minor allele frequency; het, heterozygote; homo, homozygote; n, number*.

Samples from two patients were tested for β-glucosidase enzyme level and chitoriosidase level by the use of a validated functional assay (fluorimetric assay method) (23) at the Guy's Hospital, London, United Kingdom.

### Statistical Analysis

Statistical analysis was performed using IBM SPSS Statistics, Version 22.0. Cohort characteristics were assessed using descriptive statistics. Bivariate associations between categorical variables were calculated using Pearson chi-square tests (*X*^2^) or Fisher's exact tests (when expected cell counts ≤ 5 observations, e.g., genotype frequencies comparison). Where continuous variables were normally distributed, independent sample *t*-tests were used, and where not, Mann–Whitney *U*-tests. The results were deemed statistically significant where *p* < 0.05. Two hypotheses were tested. The first was that the presence of a *GBA* variant increased the risk of PD in the Irish population. Logistic regression model was used to control for age and gender. The second hypothesis was that the *GBA* variant carriers have earlier age-at-PD onset (14). A linear regression model was fitted with age at onset as the dependent variable and the presence or absence of a *GBA* variant as the independent variable (gender as a covariate). Linear and logistic regression models were used to test whether the frequency of the motor complications, wearing off, dyskinesia, dystonia, freezing of gait, hallucinations, dementia, and Unified Parkinson's Disease Rating Score part III (UPDRS-III) differed between carriers and non-carriers. Gender, age, and disease duration were considered as covariates. In the sensitivity analysis, each of the motor complications was also controlled for medications, UPDRS-III score, and disease subtype.

## Results

We screened 314 Irish patients with PD of which 62.4% (*n* = 196) were male and 37.6% (*n* = 118) were female, with mean age at inclusion of 64.94 ± 10.69 years, mean age at onset of 56.23 ± 12.04 years, and mean disease duration of 8.62 ± 6.97 years. The majority had tremor-predominant disease (55.7%), followed by postural instability gait disorder (PIGD) (34.1%) and mixed subtypes (10.2%). Motor complications were seen in 43.3%. Family history of PD was present in 173 patients (55.1%). A group of 96 Irish controls [51% men (*n* = 49), 49% women (*n* = 47), mean age at inclusion (61.15 ± 14.5)] was studied to assess the mutation frequency in the ethnically matched population.

We examined 314 patients for genes associated with autosomal dominant PD. Among 314 patients, we identified one PD patient with LRRK2 G2019S mutation positive for *GBA* benign intronic variant (exon 7−18 bp). We did not identify any carriers of p.A53T or p.A30P in SNCA or p.D620N in VPS35. There were no patients with *SNCA* dosage alterations.

On examination of the 81 patients with YOPD, we identified one homozygote carrier (p.G430D/p.G430D), three compound heterozygote (p.Leu112fsX163/p.R275W; p.G430D/Ex 4&5del; p.R275W/Ex3 del), and three heterozygote carriers [p.R275W/wt. (*n* = 1); p.P437L/wt (*n* = 2)] in *PARKIN* gene. None of the *PARKIN* gene carriers had variants in *GBA* detected. No homozygous or compound heterozygous carriers of *PINK1* or *DJ1* were detected.

We detected 26 carriers of *GBA* pathogenic/risk variants in PD and 3 in controls [p.E326K, P.T369M, p.N370S, p.F216Y, p.D409H, and RecNcil (p.L444P-A456P-V460V)] (Tables 2, 3). These variants were found in 8.3% of PD patients and 3.1% of controls (*p* = 0.08, *X*^2^). The result remained non-significant after controlling for age and gender [odd's ratio (OR), 3.2 (*p* = 0.06; 95%CI, 0.94–10.97). RecNcil and p.N370S carrier frequency in PD was 1.9% [RecNcil, *n* = 3, minor allele frequency (MAF) = 0.48%; p.N370S, *n* = 3, MAF = 0.478%); these variants represented 18.2% (6/33) of the pathogenic/risk variants and were not present in controls. The frequency of *GBA* mutations in familial PD was 8.1% (*n* = 14) and 8.6% (*n* = 12) in sporadic PD. The most common variants detected in PD were the risk variants: p.E326K (12 heterozygotes including p.E326K/G377C and p.E326K/T369M and 1 homozygote, p.E326K/E326K) and p.T369M (*n* = 6, including mentioned above p.E326K/T369M), followed by the pathogenic variants: RecNcil (*n* = 3) and p.N370S (*n* = 3) (Tables 2–4). Additionally, we detected three variants of unknown significance (VUS), p.G195E, p.R301H, and p.R368C, in three PD patients and none in controls (Tables 1, 4). We also identified 11 carriers of benign, known intronic variants (rs140335079, T > A: three heterozygotes, five homozygotes; exon 7–17 bp, G > C: one homozygote; rs377143075, T > C: two heterozygotes).

**Table 3:** Genotype–phenotype correlation in risk variants of GBA carriers with Parkinson's disease (PD).

**ID**	**Genotype**	**Sex**	**Familial PD**	**Age (years)**	**Onset (years)**	**First symptom**	**Duration (years)**	**Subtype**	**UPDRSIII**	**H&Y**	**Cognitive decline**	**Motor**	**Hallucinations**	**FOG**
1	E326K/wt	M	–	69	62	Bradykinesia	7	PIGD	51	2	+	Dyskinesia wearing off	–	–
2	E326K/wt	M	–	64	59	Bradykinesia	5	PIGD	10	2	–	–	–	–
3	E326K/wt	M	–	51	43	Tremor	8	Tremor	15	1	–	Wearing off	–	–
4	E326K/wt	F	–	75	73	Tremor	2	Tremor	17	2	+	–	–	–
5	E326K/wt	F	+	72	50	Tremor	22	PIGD	25	3	+	Dyskinesia wearing off dystonia	+	-
6	E326K/wt	F	+	64	64	Tremor	0	Tremor	13	1	–	–		
7	E326K/wt	F	+	71	65	Pain	6	PIGD	24	2	–	Dyskinesia		
8	E326K/wt	M	+	65	43	Gait	22	Mixed	9	2	PDD	Dyskinesia wearing off	–	–
9	E326K/wt	F	+	61	56	Writing	5	Tremor	20	1	–	–	–	–
10	E326K/wt	F	+	62	58	Tremor	4	Tremor	14	1	–	–	–	–
11	E326K/E326K	F	–	65	49	Bradykinesia	16	PIGD	20	2	+	Dyskinesia	+	+
12	E326K/T369M	F	–	77	62	Tremor	15	Mixed	missing	3	PDD	Dyskinesia wearing off	+	+
13	E326K/G377C	M	+	54	39	Tremor	15	Mixed	7	1	–	Dyskinesia Wearing off	–	–
14	T369M/wt	M	–	54	44	Tremor	10	Tremor	22	2	–	–	–	–
15	T369M/wt	M	–	79	69	Tremor	10	Tremor	54	3	–	Dyskinesia wearing off	–	–
16	T369M/wt	M	–	86	61	Tremor	25	Tremor	44	2	–	–	+	–
17	T369M/wt	F	–	61	55	Tremor	6	Tremor	27	2	–	–	–	–
18	T369M/wt	M	+	38	32	Bradykinesia	6	PIGD	36	2	–	Dyskinesia wearing off dystonia	–	–

*FOG, freezing of gait; H&Y, Hoehn and Yahr score*.

**Table 4:** Genotype–phenotype correlation in GBA pathogenic variants and variants of unknown significance carriers with Parkinson's disease (PD).

**ID**	**Genotype**	**Sex**	**Familial PD**	**Age (years)**	**Onset (years)**	**First symptom**	**Duration (years)**	**Subtype**	**UPDRSIII**	**H&Y**	**Cognitive decline**	**Motor**	**Hallucinations**	**FOG**
**Pathogenic variants**													
19	F216Y/wt	M	+	56	53	Tremor	3	Tremor	28	2	–	–	–	–
20	N370S/wt	F	+	82	67	Tremor	15	Tremor	31	2	+	–	–	–
21	N370S/wt	M	–	64	61	Tremor	2.5	Mixed	46	2	PDD	–	–	–
22	N370S/wt	F	+	78	70	Tremor	8	PIGD	9	2	–	Dyskinesia	–	–
23	RecNcil	F	–	61	56	Tremor	5	Tremor	9	2	–	–	–	–
24	RecNcil	F	+	59	54	Tremor	5	PIGD	missing	2	PDD	Wearing off	+	-
25	RecNcil	F	+	33	30	Bradykinesia	3	Mixed	13	1	–	Dyskinesia wearing off dystonia	–	–
26	D409H/wt	M	+	64	60	Tremor	3.5	Tremor	24	1	–	Wearing off	–	–
**Variants of unknown significance**													
27	R368C/wt	M	–	61	55	Bradykinesia	7	PIGD	28	2	–	–	–	–
28	R301H/R301H	M	–	59	57	Bradykinesia	2.5	PIGD	37	3	–	–	–	–
29	G195E/wt	M	+	69	54	Tremor	15	Tremor	28	1	+	–	–	–

*FOG, freezing of gait; H&Y, Hoehn and Yahr scale*.

The mean age at onset for the mild mutation carriers (p.N370S *n* = 3) was 66 (±4.58) years, while for the severe mutations (p.D409H, *n* = 1; RecNcil, *n* = 3), it was 50 (±13.56) years (*p* = 0.11, independent *t*-test). The mean age at onset for the risk variants carriers (p.E326K, *n* = 12; p.T369M, *n* = 6 including p.E326K/p.T369M) was 54.66 (±11.15).

**Figure 1:**
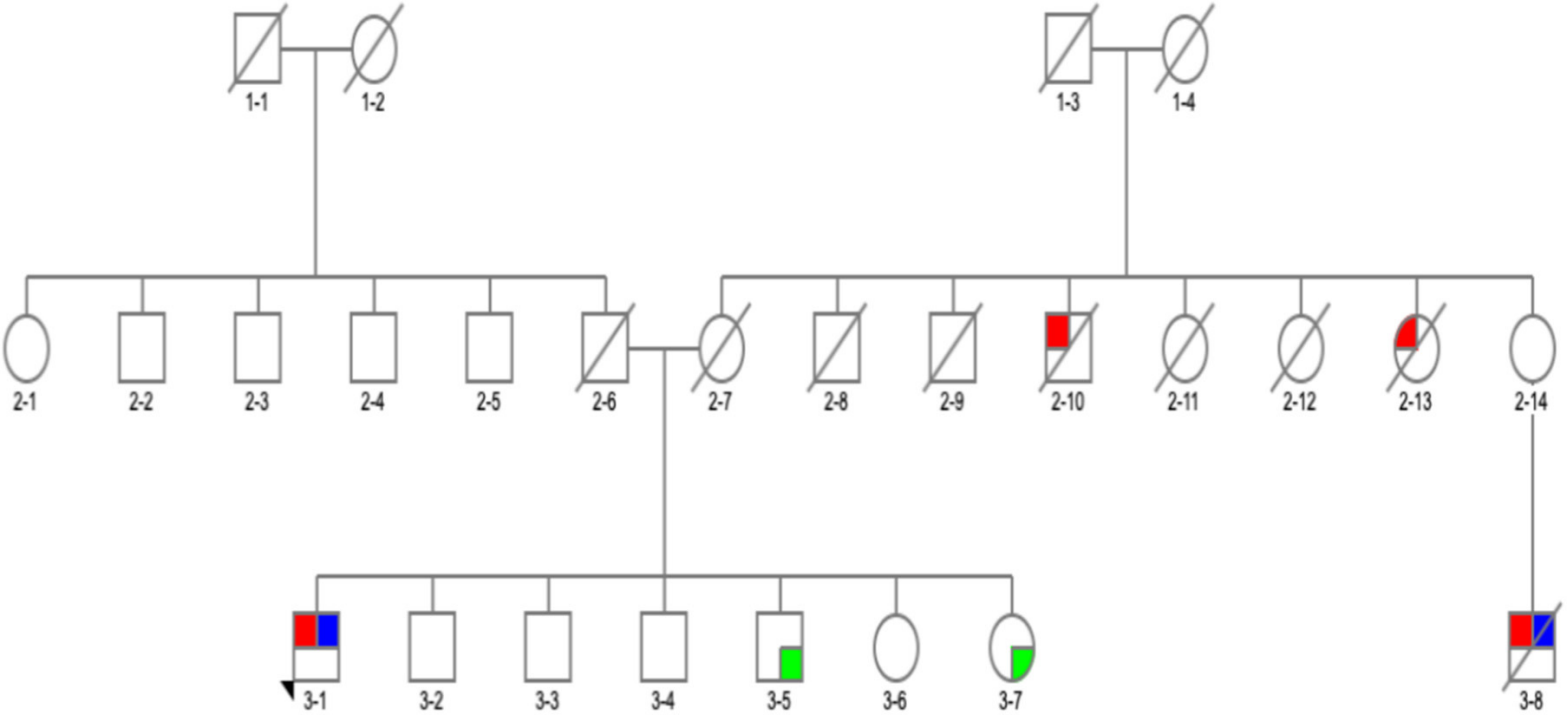
Family pedigree of the patient with *GBA* p.G195E variant in exon 6, rs74462743: red, affected with Parkinson's disease (PD); blue, tested and positive for p.G195E variant; green, tested and negative for p.G195E variant; arrow, proband; diagonal line, deceased.

We found a VUS p.R301H variant, which has not been previously reported either in ExAC (24) or gnomAD (24) databases in a homozygous state. The affected was a man with PIGD from age 57 years, normal cognition, and Hoehn and Yahr (H&Y) stage 3 who was asymptomatic for Gaucher's disease. We detected two very interesting variants. p.G195E reported in Gaucher's disease, but not in PD, predicted to be pathogenic by *in silico* tools (25) in a 69-year-old man. β-Glucosidase enzyme level was measured, and it was at 10.3 nmol/h/mg protein (normal range, 8.4–32.8), and plasma chitoriosidase level was normal. The patient had unilateral, tremor-predominant, levodopa-responsive PD and normal cognition from age 54 years (Figure 13-1). Later on, his cognition declined (MoCA score was 25/30: visuospatial/executive abilities and delayed recall were affected), but hallucinations were not present. He had mild drooling of saliva during the nighttime and REM sleep behavior disorder (RBD). His medications included carbidopa/levodopa 25/100 mg four times daily, mirabegron 50 mg for urinary frequency, and citalopram 15 mg for well-controlled depression. He was also on a continuous positive airway pressure (CPAP) machine for an obstructive sleep apnea. His MRI brain was normal, and dopamine transporter single photon emission computerized tomography (DaT scan) showed a decreased dopamine tracer uptake more pronounced on the left side of the brain. On examination he was hypophonic, had slight rigidity in all limbs, and was bradykinetic more on the right than on the left side. He had bilateral postural and kinetic hand tremor without rest tremor. He did not have any other motor complications.

There was a PD history in his maternal aunt (deceased) (Figure 12-13), maternal uncle (deceased) (Figure 12-10) and in his 67-year-old cousin (deceased) (Figure 13-8). He had tremor-predominant PD since age 51 years and was positive for the same variant. Initially, he was started on mirapexin for 2 years. He developed hallucinations at age 56 (benign, mainly animals) treated with quetiapine 25 mg in the morning and 50 mg at night. He became forgetful, developed dementia (MoCA score, 7/30), and was commenced on donepezil 10 mg once a day and memantine 10 mg twice a day. He then was switched to carbidopa/levodopa preparation 25/100 mg two tablets three times daily. His other non-motor symptoms included depression—stable on venlafaxine (effexor XL) 150 and 75 mg, and RBD. He was unable to turn in bed and get out of a chair without help and had freezing of gait episodes. There was no wearing off or dyskinesia present. He needed assistance with cutting food, dressing up, and hygiene. His swallow became affected with occasional choking episodes; therefore, he was commenced on thickened fluid diet. He had a urinary catheter *in situ* due to the difficulties arising from the urinary urgency. The biggest problem was agitation in the evening. At the time of recruitment, he was wheelchair bound, and his UPDRS-III score was 28. On examination, there was slight, however ongoing, resting tremor and bilateral, severe bradykinesia, and rigidity. The dopamine uptake on the DaT scan at age 65 was reported as profoundly decreased. The variant was absent in two asymptomatic siblings of the proband age 57 and 67 (Table 3). Neurological examination was normal in both cases, and MoCA test was 30/30 in the sister (**Figure 1**3-7) and 29/30 in the brother (Figure 13-5). There were no subtle signs of Gaucher's disease with normal hematology, biochemistry, and liver tests.

The second interesting variant found was p.G377C (c.1246 G > T) variant (not reported in databases), predicted to be pathogenic by *in silico* tools (Tables 1, 2) in a 54-year-old man (Figure 23-1). On further analysis, the β-glucosidase enzyme level was 10.8 nmol/h/mg protein (normal range, 8.4–32.8). The enzyme level in simultaneous controls was 13.6, 14.2, and 14.4. The level of plasma chitotriosidase was normal (as expected in a heterozygous asymptomatic for Gaucher's disease patient). The patient had tremor-predominant PD and normal cognition since age 39 years. There were no hallucinations. At 42 years old, he developed dyskinesia, which was treated by a deep brain stimulator (DBS) at age 49. He also had dystonia and micrographia (the majority of the words was not legible when written). At the time of recruitment, he was on amantadine 100 mg twice a day, selegiline 5 mg, slow release levodopa preparation at night (half-sinemet CR), and carbidopa/levodopa 50/12.5 mg five times a day. On examination, there was hypophonia, moderately stooped posture, and slow gait. PD was present in his mother diagnosed at age 45 (Figure 22-6), who died at 73, maternal aunt diagnosed in her 70s (deceased at 78) (Figure 22-9), and maternal grandfather (Figure 21-3).

**Figure 2:**
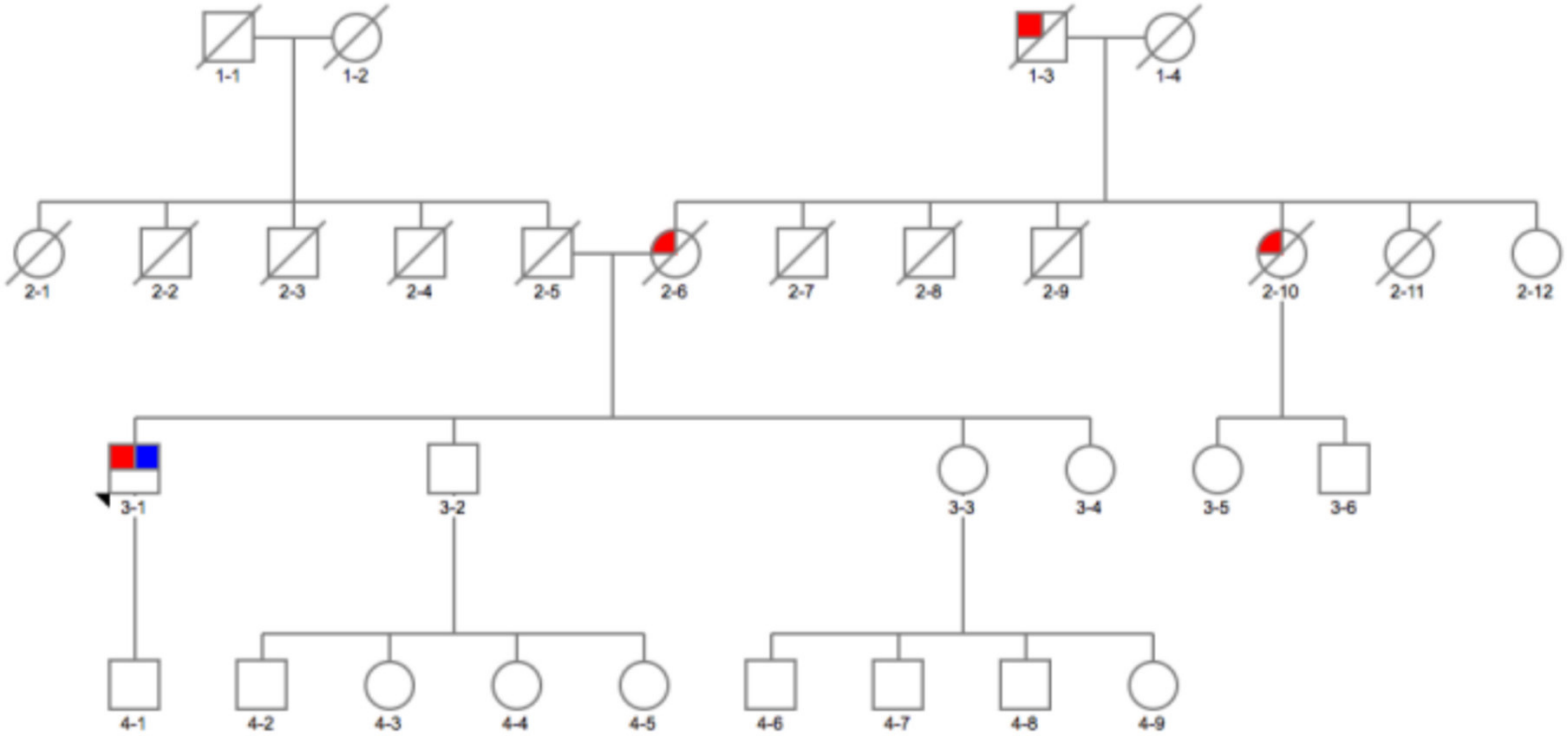
Family pedigree of the patient with *GBA* p.E326K variant in exon 8 and p.G377C variant in exon 9: red, affected with Parkinson's disease (PD); blue, tested and positive for p.E326K/G377C variant; arrow, proband; diagonal line, deceased.

On genotype-phenotype assessment between 26 mutation/risk variant carriers and 285 non-carriers, the median age at onset in both groups was the same (57 years), carriers were more likely to be a female (53.8% carrier women vs. 46.2% carrier men, *p* = 0.08, *X*^2^), and the majority (65.4%) of carriers had late onset PD (*n* = 17, *p* = 0.31, *X*^2^) and reported having a positive PD family history (53.8%, *n* = 14, *p* = 0.87, *X*^2^) (Table 5). However, these results were statistically non-significant (Table 1). Cognitive problems were present in 34.6% of carriers in comparison to 29.8% in non-carriers; however, the *p*-value was non-significant. Hallucinations were four times more prevalent in carriers than non-carriers [*p* = 0.01, OR 3.97 (95%CI 1.434–11.015), Fisher's exact test]. This remained true after adjusting for age, gender, disease duration, dementia, cognitive impairment, and medications. While additionally controlled for the presence of *Parkin* homozygous/compound heterozygous or *LRRK2* mutations, the prevalence of hallucinations in *GBA* mutation carriers remained significantly higher than in *GBA* non-carriers [*p* = 0.04, OR 4 (95%CI 1.1–15.3)]. In terms of motor complications dyskinesia [*p* = 0.003, OR 3.36 (95%CI 1.46–7.75, *X*^2^ test)] and dystonia [*p* = 0.009, OR 12.261 (95% CI 2.34–64.212, Fisher's exact test)] were more prevalent in carriers vs. non-carriers and were independently associated with the carrier status after controlling for age, gender, disease duration, medications, UPDRS III score, and subtypes. When the presence of dyskinesia and dystonia was controlled for the presence of *PARKIN* homozygous/compound heterozygous or *LRRK2* mutations, the result for dyskinesia remained significant [dyskinesia: *p* = 0.007, OR 4.37 (95%CI 1.49–12.85)], but it became non-significant for dystonia (*p* = 0.088). There was no association found between other variables and *GBA* status (Table 5).

**Table 5:** Genotype–phenotype assessment in GBA pathogenic/risk variants carriers with Parkinson's disease.

	**GBA pathogenic and risk variants carriers *n* = 26**	**Non-carriers *n* = 285**	***p*-value**
Gender	*n* (%)	*n* (%)	
Women	14 (53.8%)	104 (36.5%)	
Men	12 (46.2%)	181 (63.5%)	0.08^a^
	Median (range)	Median (range)	
Age at assessment	64 (33–86)	67 (32–89)	0.58^c^
Age at onset	57 (30–73)	57 (16–85)	0.68^c^
	*n* (%)	*n* (%)	
Early onset PD <50	7 (26.9%)	74 (26%)	0.91^a^
	Median (range)	Median (range)	
Disease duration	6 (0–25)	7 (0.5–50)	0.95^c^
Family history	*n* (%)	*n* (%)	
Yes	14 (53.8%)	158 (55.4%)	
No	12 (46.2%)	127 (44.6%)	0.88^a^
Cognition	*n* (%)	*n* (%)	
MCI-PD	5 (19.2%)	54 (18.9%)	1^b^
PDD	4 (15.4%)	31 (10.9%)	0.51^b^
Hallucinations	6 (23.1%)	20 (7%)	**0.01**^b^
Motor complications	14 (53.8%)	122 (42.8%)	0.27^a^
Wearing off	11 (42.3%)	92 (32.3%)	0.29^a^
Dyskinesia	11 (42.3%)	51 (17.9%)	**0.003^a^**
Dystonia4	3 (11.5 %)	3 (1.1%)	**0.009**^b^
Freezing of gait	3 (11.5%)	31 (10.9%)	1^b^
DBS *in situ*	1 (3.8%)	6 (2.1%)	1^b^
	*n* = 24	*n* = 278	
	Median (range)	Median (range)	
UPDRS-III score	21 (7–54)	22 (3–83)	0.18^c^
UPDRS-III categories	*n* (%)	*n* (%)	
<32 (mild)	19 (79.2%)	224 (80.6%)	
33–58 (moderate)	5 (20.5%)	52 (18.7%)	
> 58 (severe)	0 (0%)	2 (0.7%)	0.1^d^
Falls	3 (11.5%)	25 (8.8%)	0.71^b^
Medications	*n* (%)	*n* (%)	
Levodopa	22 (84.6%)	230 (80.7%)	0.79^b^
MAOBI	16 (61.5%)	169 (59.3%)	0.82^a^
Dopamine agonists	11 (42.3%)	131 (46%)	0.72^a^
Madopar			
(levodopa+benserazide)	1 (3.8%)	26 (9.1%)	0.7^b^
Anticholinergics	1 (3.8%)	23 (8.1%)	0.51^b^
Amantadine	2 (7.7%)	28 (9.8%)	0.76^b^
Duodopa	0 (0%)	4 (1.4%)	1^b^
Apomorphine	1 (12.5%)	7 (2.5%)	1^b^
Subtype			
Tremor-predominant	13 (50%)	161 (56.5%)	0.52^a^
PIGD	8 (30.8%)	97 (34%)	0.73^a^
Mixed	5 (19.2%)	27 (9.5%)	0.16^b^
First symptom		
Tremor	18 (69.2%)	190 (66.7%)	0.79^a^
Bradykinesia	5 (19.2%)	59 (20.7%)	0.85^a^
Stiffness	0 (0%)	6 (2.1%)	0.67^b^
Writing difficulties	1 (3.8%)	5 (1.8%)	1^b^
Pain	1 (3.8%)	1 (0.4%)	0.16^b^
Speech problems	0 (0%)	2 (0.7%)	1^b^
Gait problems (all)	1 (3.8%)	22 (7.7%)	1^b^
Loss of arm swing	0 (0%)	3 (1.1%)	1^b^
Shuffling	0 (0%)	12 (4.2%)	0.4^b^
Dragging a leg	1 (3.8%)	6 (2.1%)	1^b^
Balance problems	0 (0%)	1 (0.4%)	1^b^

a *Pearson chi square test*,

b *Fisher exact test*,

c *Mann–Whitney U-test*,

d *Linear by linear Armitage exact trend test*.

*Bold value indicates statistically significant*.

## Discussion

*GBA* mutations carried in the heterozygous state are a strong risk factor for developing α-synucleinopathy including PD. *GBA* variants also appear to act as phenotypic modifiers affecting cognition and motor progression of PD. The frequency of *GBA* variants differs across populations (9, 26, 27), and herein, we show that patients with PD in Ireland have a relatively high frequency of *GBA* mutations (PD, 8.3%; controls, 3.1%). The frequency is higher than that of the Portuguese (6.1%, 14/230) (28), Greek (4.7%, 8/172) (29), Flanders–Belgian population (4.5%, 12/266) (30), and British (4.2%, 33/790) (10), and slightly above the average European carrier frequency (6.7%, 76/1,130) (16). The carrier frequency both in Irish patients and controls was high when compared to that reported in most European studies with the exception of Spain (9.8%, 22/225) (31). The higher *GBA* frequency in Spain could be related to the higher rate of Jewish ancestry in the Iberian Peninsula (32).

While the number of p.N370S and RecNcil carriers was equal in Irish PD (with no carriers of p.L444P found), it has been reported that p.N370S is more prevalent in the Europeans and Ashkenazi Jews (70%), and p.L444P is the most common mutation in Chinese (62%) (27, 33). It could be argued that p.T369M should not be included in our pathogenic/risk group, as the number of p.T369M carriers was greater in the control group p.T369M (*n* = 6) than in the PD group (*n* = 5 heterozygotes and *n* = 1 compound heterozygote with p.E326K); however, we did include p.T369M variant in the pathogenic/risk group in agreement with the most recent literature (4, 6, 14, 15).

We detected a p.G377C (p.G416C, c.1246G > T) variant. The G > T nucleotide change in position 416 has not been found in the available databases; however, a change G > A (p.G416S, rs121908311) in the same position is classified as pathogenic. The β-glucosidase enzyme level in a heterozygote carrier within the normal range found in our patient is consistent with the report by Alcalay et al. (26) (p.E326K does not cause Gaucher's disease even in a homozygote state, and the enzymatic level is also within the normal range) (26). The p.G377C variant was described in one case study of Gaucher's disease from Northern Ireland (34) as a compound heterozygote, but not in PD. Only homozygous or compound heterozygous variants cause Gaucher's disease; therefore, p.G377C was necessary to contribute to the disease in the case report by Illingworth (34). Moreover, the level of plasma chitotriosidase was 8,000 in the Illingworth (34) case, further supporting symptomatic Gaucher's disease (the level of chitotriosidase is only elevated in symptomatic patients with Gaucher's disease). Taken together, these evidence support the likely pathogenicity of this variant.

We also identified a p.G195E variant implicated in Gaucher's disease (25) that cosegregated with PD, which merits further investigation (the at-risk siblings are now older than the affected, but we cannot exclude the development of PD at a later age due to the intrafamilial PD heterogeneity). This finding may suggest a more significant role of p.G195E in PD; however, this variant needs to be further explored.

There was no association of gender with pathogenic/risk variants in PD in keeping with findings from Lesage et al. where there was no difference detected (9). Male/female ratio was also reported 5:1–3:2 in other studies (35). Combined MCI-PD (*n* = 5/26) and PDD (*n* = 4/26) occurred in 34.6%. This is in keeping with other studies (24–48%) (36). The more common cognitive decline (six-fold increased dementia risk) has been reported in the literature (5, 6, 31), but the prevalence of cognitive impairment and dementia analyzed either separately or as one group in our study did not differ between carriers and non-carriers (however, the sample was small). The *GBA* mutation/risk variant presence was independently associated with hallucinations in our study in keeping with other studies (31). The three-fold higher occurrence of dyskinesia in the *GBA* carrier group in comparison to non-carriers in our study is in keeping with other reports. The higher occurrence of dyskinesia suggests that *GBA* carriers may be more sensitive to medications and genetic assessment in the appropriate patient should be taken into consideration (5, 9, 36) (similarly to the levodopa sensitivity and more frequent dyskinesia resulting in small levodopa doses being used for parkin mutations carriers). While the number of patients with YOPD in the study (*n* = 81) may seem high, and this group is interesting in itself from the genetic perspective, these were neither specifically preselected for the recruitment nor were they related in any way (only probands were reported in this study).

There are several strengths of our study including the examination of the *GBA* prevalence in Irish PD for the first time, the full sequencing of coding regions (and exon-intron boundaries) of the *GBA* gene on the 3.14% of the Irish PD population (314 PD patients recruited/10,000 number of PD patients in Ireland) (limited screening for p.N370S and p.L444P would result in 81.8% of the mutations being missed in our study), comparison to the ethnically matched control group with a high genotyping success rate, and comprehensive investigation of other causes of autosomal dominant and recessive PD (making this study the most comprehensive and up-to-date report of genetics of PD in the Irish population).

Our study also has a few limitations: it was an observational cross-sectional study; therefore, the progression of the disease could not be assessed, and a longitudinal study should be performed in the future. It should be noted that we were unable to reliably determine phase for the two individuals with a presumed compound heterozygous carrier state, both carried the risk variant E326K (E326K/T369M, *n* = 1; E326K/G377C, *n* = 1); however, these subjects would fall into the risk/pathogenic group regardless of being in “cis” or in “trans” status. While we acknowledge the small sample size of the control group in comparison to the PD group most likely leading to the non-significance of the *GBA* prevalence data, in our opinion, having a control group is an important part of any *GBA* study.

With the advent of new screening technologies, *GBA* carriers will be encountered more frequently in our clinics, and it will be essential to prepare a mutation-specific approach to the management of PD. Efforts to find a disease-modifying treatment for *GBA* carriers with PD are ongoing. There are currently two clinical trials underway, one trial examining ambroxol, an over-the-counter medication used to reduce mucus production in respiratory tract disorders, and its influence on motor and cognitive progression in *GBA* carriers, and a placebo controlled phase 2 double-blind study (MOVES-PD) of GZ/SAR4027671—a molecule capable of crossing brain-blood barrier (37, 38). Another recently completed trial showed that the target engagement and CSF penetration were accomplished in PD patients treated with oral ambroxol. While the CSF glucocerebrosidase activity decreased, the glucocerebrosidase protein levels and alpha-synuclein levels increased and UPDRS-III score improved by a mean of 6.8 points. Notably, these changes occurred both in patients with and without *GBA* mutations (39). There are no recommendations of how to proceed, where the risk of PD is disclosed, but preventative approaches may be rapidly approaching. Patients should be informed about the increased possibility of cognitive decline, depression, falls, autonomic vulnerability, family (35) planning, and disease progression [carriers are reported to die earlier at 75.7 years (SD 5.5) than non-carriers 80.9 (6.6)] (40). Treatment options for *GBA* mutation carriers should be a little bit distinct and focus on the avoidance of medications increasing the risk of falls (lowering blood pressure), worsening cognitive status, and deferral of levodopa therapy (36). Further research is required, but personalized therapeutic approach for PD may be closer than we might think.

## Conclusion

We present the most comprehensive and up-to-date overview of genetics of PD in the Irish population. We, for the first time, showed the link between *GBA* and PD in Ireland. In agreement with our hypothesis, the findings of our study suggest that the *GBA* prevalence in PD is higher than in most European countries, and genetic background of Irish PD patients warrants further studies.

## Data Availability Statement

The raw data supporting the conclusions of this article will be made available by the authors, without undue reservation.

## Ethics Statement

The studies involving human participants were reviewed and approved by Mater Misericordiae University Hospital, Dublin, Ireland 1/378/1300. The patients/participants provided their written informed consent to participate in this study. Written informed consent was obtained from the individual(s) for the publication of any potentially identifiable images or data included in this article.

## Author Contributions

DO: study idea and design, study recruitment and phenotypic assessment, DNA extraction, GBA sequencing, and laboratory work, interpretation of the results and statistical analysis, preparation of the first draft of manuscript, correction, and approval of the final draft. AM: study recruitment and phenotypic assessment, DNA extraction, critique, and approval of the final draft. AS-B and RW: laboratory work, critique, and approval of the final draft. BM: study recruitment, critique, and approval of the final draft. RM and OH: critique and approval of the final draft. OR: study idea, lead of the laboratory work and expertise, overlooking the laboratory work at the Mayo Clinic, expertise in the interpretation of the results, critique, and approval of the final draft. TL: study idea and design, lead of the neurological expertise, overlooking the study in the Dublin Neurological Institute, critique, and approval of the final draft. All authors: read and approved the final version on the manuscript.

## Conflict of Interest

The authors declare that the research was conducted in the absence of any commercial or financial relationships that could be construed as a potential conflict of interest.
