# Supplementary material for: Association Between Glucocerebrosidase Mutations and Parkinson's Disease in Ireland
Source: Front Neurol. 2020 Jun 30;11:527. doi: 10.3389/fneur.2020.00527 (PMC7344206; doi:10.3389/fneur.2020.00527)
Supplement: Supplementary file 1 [file Table_1.DOCX]

**Table 1 *GBA* PCR primer sequences and PCR conditions**

TD: Touchdown PCR; Q: A reaction additive supplied from Qiagen that allows the efficient amplification of “difficult‟ (e.g. GC-rich) templates.

| *GBA* |  | PCR primer sequence  5'-Forward-3' | PCR primer sequence  5'-Reverse-3' | PCR  Conditions  All with Q |
| --- | --- | --- | --- | --- |
| Amplicon 1 | exon 1-5 | CCTAAAGTTGTCACCCATAC | AGCAGACCTACCCTACAGTTT | 65-50 TD (40 Long range) |
| Amplicon 2 | exon 5-7 | GACCTCAAATGATATACCTG | AGTTTGGGAGCCAGTCATTT | 60-55 TD (Long range) |
| Amplicon 3 | exon 8-11 | TGTGTGCAAGGTCCAGGATCAG | ACCACCTAGAGGGGAAAGTG | 65-55 TD (Long range) |

**Table 2 *GBA* sequencing primers**

| *GBA* | Sequencing primers sequence  5'-Forward-3' | Sequencing primers sequence  5'-Reverse-3' |
| --- | --- | --- |
| exon 1 | CCTAAAGTTGTCACCCATAC | CCCTCCATCTGTGCCTTGCTC |
| exon 2 | GAGAGTAGTTGAGGGGTGGA | CAAAGGACTATGAGGCAGAA |
| exon 3 | ATGTGTCCATTCTCCATGTC | GGTGATCACTGACACCATTT |
| exon 4 | GGTGTCAGTGATCACCATGG | ACGAAAAGTTTCAATGGCTCT |
| exon 5 | GCAAGTGATAAGCAGAGTCC | AGCAGACCTACCCTACAGTTT |
| exon 6 | CTCTGGGTGCTTCTCTCTTC | ACAGATCAGCATGGCTAAAT |
| exon 7 | TTGGCCGGATCATTCATGAC | AGTTTGGGAGCCAGTCATTT |
| exon 8 | TGTGTGCAAGGTCCAGGATCAG | TTTGCAGGAAGGGAGACTGG |
| exon 9 | CACAGGGCTGACCTACCCAC | GCTCCCTCGTGGTGTAGAGT |
| exon 10 | CAGGAGTTATGGGGTGGGTC | GAGGCACATCCTTAGAGGAG |
| exon 11 | GTGGGCTGAAGACAGCGTTGG | ACCACCTAGAGGGGAAAGTG |
